# The Relationship between Atherogenic Index of Plasma and Obesity among Adults in Taiwan

**DOI:** 10.3390/ijerph192214864

**Published:** 2022-11-11

**Authors:** Jen-Shan Zhang, Wei-Chung Yeh, Yi-Wen Tsai, Jau-Yuan Chen

**Affiliations:** 1 Cathay General Hospital, Taipei 106, Taiwan; 2 Department of Family Medicine, Chang Gung Memorial Hospital, Keelung 204, Taiwan; 3 College of Medicine, Chang Gung University, Taoyuan 333, Taiwan; 4 Department of Family Medicine, Chang Gung Memorial Hospital, Linkou Branch, Taoyuan 333, Taiwan; 5 Department of Family Medicine, New Taipei Municipal TuCheng Hospital (Built and Operated by Chang Gung Medical Foundation), New Taipei City 236, Taiwan

**Keywords:** atherogenic index of plasma, cardiovascular diseases, obesity, BMI, hospital employees

## Abstract

Atherogenic index of plasma (AIP), a novel biomarker, is associated with cardiovascular diseases and obesity. The main aim of this study was to investigate the relationship between AIP and obesity among Taiwanese hospital employees. A total of 1312 subjects with an average age of 42.39 years were enrolled in this cross-sectional study. AIP was calculated as log10 (TG/HDL-C). All subjects were divided into three groups according to AIP tertiles. Chi-square test, independent *t*-test and one-way ANOVA were used to compare the demographic and clinical lab characteristics of the three groups. Multivariate logistic regression analysis was used to assess the relationship between AIP and obesity. The results showed that subjects with obesity or with high AIP levels exhibited significant differences in systolic blood pressure, diastolic blood pressure, waist circumference, alanine aminotransferase, fasting plasma glucose, high-density lipoprotein cholesterol, low-density lipoprotein cholesterol, triglycerides and prevalence of diabetes mellitus, hypertension, hyperlipidemia and metabolic syndrome. In addition, age and total cholesterol were increased in the high AIP group. Increased AIP levels were strongly associated with obesity.

## 1. Introduction

The prevalence of obesity has increased worldwide during the past 50 years, reaching pandemic levels [1]. Obesity has become a global health problem because it significantly increases the risk of diseases such as type 2 diabetes mellitus (DM), hypertension (HTN), cardiovascular diseases (CVD), osteoarthritis, dementia, fatty liver disease, obstructive sleep apnea and several cancers [1], thus negatively impacting both quality of life and life expectancy.

Obesity is a medical condition wherein adipocytes exhibit hypertrophy and visceral adipose tissue accumulates because calorie intake exceeds expenditure. Adipose tissue is mainly composed of lipids, including triglycerides and free cholesterol [2]. The main products secreted from adipose tissue are free fatty acids, and sustained increases in circulating free fatty acids contribute to metabolic diseases [2,3]. To date, most studies have reported that obesity and blood lipids are strongly related [4,5]. Therefore, lipid components in circulation are easily available biomarkers to predict obesity and other metabolic diseases.

According to a cohort study from 2007 to 2016 in Taiwan, the prevalence of overweight and obesity were 21% and 15%, respectively [6]. The prevalence of metabolic syndrome in Taiwanese hospital workers was not lower compared to other occupations according to a previous Taiwanese study [7]. Despite more knowledge about health issues, hospital workers in Taiwan are still at risk of obesity and metabolic syndrome.

Some studies have found that atherogenic index of plasma (AIP), a novel biomarker calculated by high-density lipoprotein cholesterol and triglycerides, is useful to predict the risk of CVD, dyslipidemia and metabolic diseases [8,9,10,11,12]. Recently, Zhu X et al. found that AIP is also strongly related to obesity [5]. However, published studies about relations between AIP and obesity in Taiwan are limited. Therefore, this study aimed to assess the association between AIP and obesity among hospital employees in a Taiwanese population.

## 2. Materials and Methods

### 2.1. Study Design and Participants

This is a retrospective and cross-sectional study. In total, 1538 subjects were recruited from annual health checkup of employees from Chang Gung Memorial Hospital between 2014 and 2018. After excluding 42 pregnant women and 184 persons with incomplete or extreme data, a total of 1312 subjects were finally included in this research. The study protocol was approved by the Chang Gung Medical Foundation Institutional Review Board and the number of Review Board was 201900263B0.

### 2.2. Data Collection

Collected data included age, sex, systolic blood pressure (SBP), diastolic blood pressure (DBP), height, and weight and waist circumference (WC). Blood biochemical analysis included alanine aminotransferase (ALT), fasting plasma glucose (FPG), high-density lipoprotein cholesterol (HDL-C), low-density lipoprotein cholesterol (LDL-C), total cholesterol (TC) and triglyceride (TG). Hypertension (HTN) was defined as self-reported HTN, systolic BP (SBP) ≥ 140 mm Hg, diastolic BP (DBP) ≥ 90 mm Hg, or the use of medications for HTN. Diabetes mellitus (DM) was defined as self-reported DM, fasting plasma glucose ≥ 126 mg/dL or the use of oral antidiabetic drugs or insulin. Dyslipidemia was defined as self-reported dyslipidemia, LDL-C ≥ 130 mg/dL, HDL-C < 40 mg/dL in men, HDL-C < 50 mg/dL in women, TG ≥ 150 mg/dL, total cholesterol ≥ 200 mg/dL, or the use of lipid-lowering medications. Metabolic syndrome was defined according to the modified criteria of the National Cholesterol Education Program Adult Treatment Panel III (NCEP-ATP III), but the standard of WC has been adjusted by the Minister of Health and Welfare of Taiwan. Therefore, metabolic syndrome is defined by the presence of three or more of the following components: (1) WC ≥ 90 cm for men and ≥ 80 cm for women; (2) TG ≥ 150 mg/dL; (3) HDL-C < 40 mg/dL for men and < 50 mg/dL for women; (4) BP ≥ 130/85 mm Hg or current use of antihypertensive medications; and (5) FPG ≥ 100 mg/dL or current use of oral antidiabetic drugs. All participants fasted for 8–12 h before blood was drawn for biochemical examinations.

### 2.3. Definition of AIP and Obesity

Atherogenic index of plasma (AIP) is calculated as log (TG/HDL-C). Body mass index (BMI) is calculated as weight (in kilograms)/height (in meters)^2^, and obesity is defined as BMI ≥ 27 kg/m^2^ according to Minister of Health and Welfare in Taiwan.

### 2.4. Statistical Analysis

SPSS 22.0 was used for statistical analyses in this study. To compare baseline variables, all subjects were divided by two methods. First, all participants were separated into obesity and non-obesity groups. In addition, all participants were divided equally into 3 subgroups according to their AIP values. Specifically, one-third of patients with the lowest AIP values were placed into one group, one-third of patients with the highest AIP were placed into another group, and the other one-third of patients comprised the middle AIP group. Independent *t*-test, one-way ANOVA, and chi-squared test were used to compare baseline variables. The correlations among BMI, AIP and four lipid components (HDL-C, TC, TG, LDL-C) were expressed by the Pearson coefficient. All statistical tests were two-sided, and a *p*-value < 0.05 was considered significant.

The relations among AIP, conventional lipid components and obesity were analyzed by univariate and multivariate logistic regression analyses. All subjects were divided into three groups based on their tertiles of AIP. Unadjusted and adjusted odds ratios (ORs) and 95% confidence intervals (95% CIs) were calculated to identify independent risk factors of obesity.

Area under curve (AUC) values of receiver operating characteristic curves (ROC curve) were calculated to compare the predictive value between AIP and three lipid components (TC, TG, and LDL-C) for predicting obesity.

## 3. Results

Among 1312 subjects enrolled for analysis in this study, 323 were obese (BMI ≥ 27), and 501 were male. The mean age was 42.39 ± 8.90 years. As shown in Table 1, obese individuals were more likely to exhibit increased SBP, DBP, WC, ALT, FPG, LDL-C and TG and reduced HDL-C. In addition, obese individuals exhibited an increased prevalence of HTN, DM, hyperlipidemia and metabolic syndrome. In Table 2, subjects with higher AIP tended to be older and male and exhibited increased SBP, DBP, WC, ALT, FPG, LDL-C, TC, TG, HTN, DM, and hyperlipidemia and reduced HDL-C.

**Figure 1:**
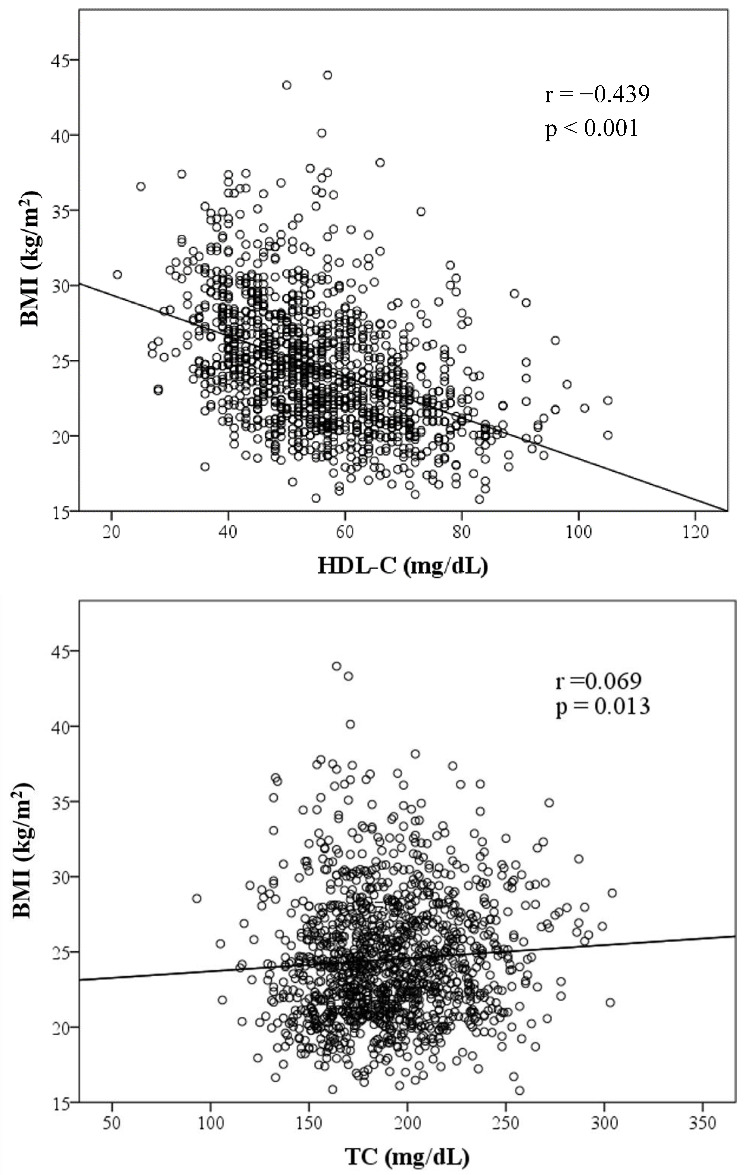

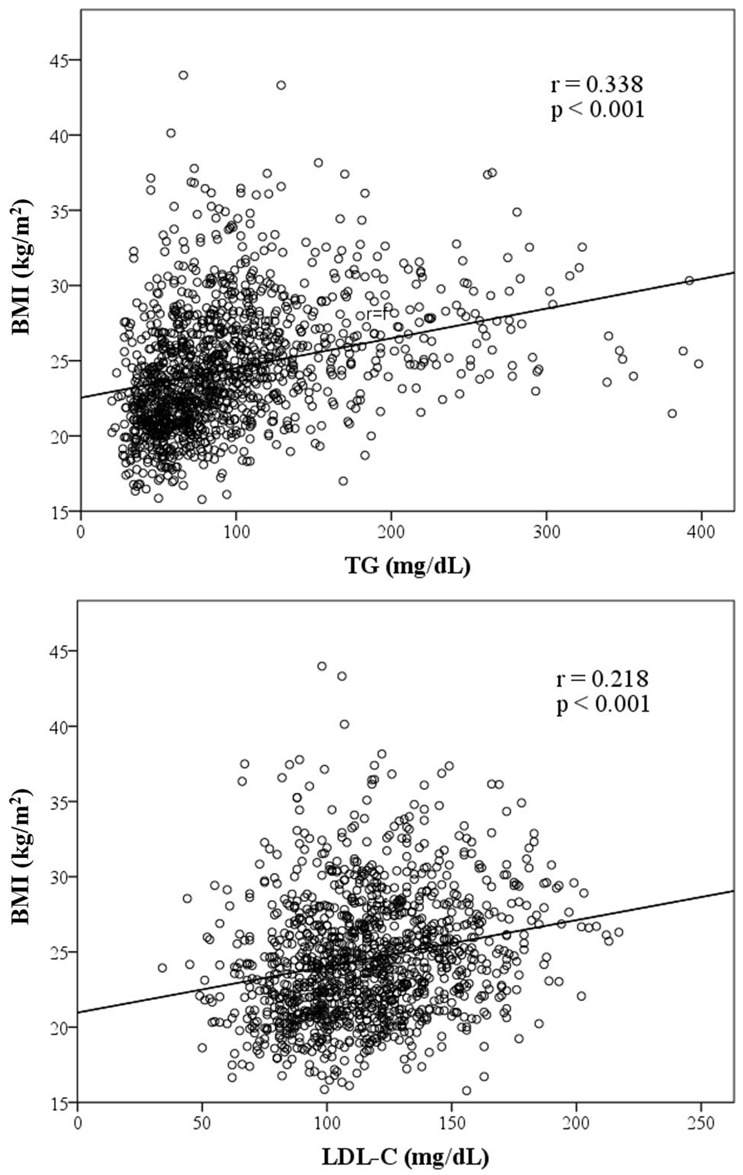
Scatter plots showing significant correlations between BMI and four lipid components, AIP. (Note: r = correlation coefficient).

Figure 1 presents graphs illustrating the association between BMI and variables of lipid components. TC, TG, LDL-C and AIP are positively correlated to BMI, whereas HDL-C is inversely correlated to BMI. HDL-C, TG, LDL-C and AIP were significantly associated with BMI. Among those factors with a positive slope, AIP exhibited the strongest association with BMI with a coefficient of 0.462 (*p* < 0.001).

Table 3 presents the results of logistic regression analysis. Univariate regression analysis showed that sex, HTN, DM, HDL-C, TC, TG, LDL-C and AIP were strongly related to obesity. For example, there was 1% increase in the odds of being obesity for 1 mg/dl increase in TG level (OR = 1.01). Besides, after adjustment by age, sex, HTN, DM, HDL-C, TC, TG, LDL-C, AIP level remained significantly associated with obesity. The odds of obesity for in high AIP level over the odds of obesity in low AIP level was 3.22. (OR = 3.22, *p* < 0.001).

**Figure 2:**
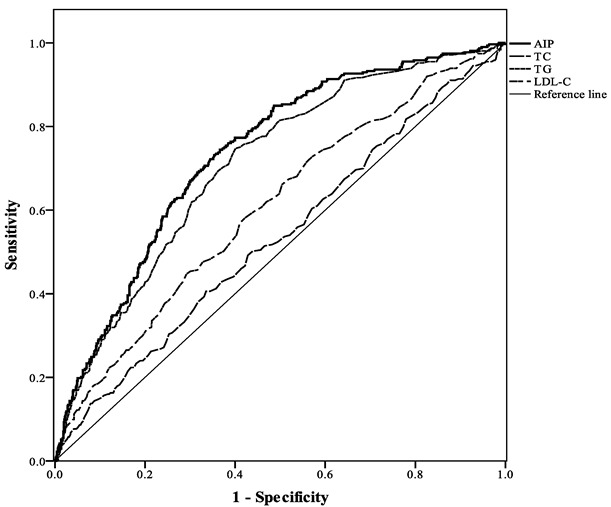
Comparison of the predictive performance of three lipid components and AIP for the presence of obesity.

As shown in Figure 2 and Table 4, AUC values of ROC were calculated to compare the predictive value between AIP and three lipid components (TC, TG, LDL-C) for predicting obesity. TG, LDL-C and AIP exhibited statistical significance. Among them, AIP demonstrated the highest predictive power with an AUC of 0.74 (the AUC values of TC, TG, and LDL-C were 0.53, 0.71, and 0.61, respectively).

## 4. Discussion

In this cross-sectional study, we explored the relationship between AIP and obesity risk. We found that subjects with higher AIP levels tended to have an increased risk of obesity. Multivariate logistic regression analysis results showed that AIP was an independent risk factor for obesity (OR = 3.22, *p* < 0.001) after adjustment by age, sex, HTN, DM, HDL-C, TC, TG, and LDL-C. We also found that AIP exhibited better predictive value for obesity with a higher AUC (0.718) than conventional lipid components, including TC, TG and LDL-C. AIP potentially represents a better biomarker for obesity.

The atherogenic index of plasma (AIP), a logarithmic transformation of the ratio of TG/HDL, is a simple biomarker proposed by Dobiasova and Frohlich in 2000. Initially, AIP or TG/HDL were used to predict the risk of cardiovascular disease or metabolic disease. For example, in 2003, T. Mclaughlin found that the TG/HDL-C might be useful to identify overweight individuals with insulin resistance [13]. Another study by T. Mclaughlin in 2005 found that insulin resistance and hyperlipidemia could be identified in patients who were at high risk of cardiovascular disease, while TG/HDL was higher than 3.5. However, this study also indicated that the specific value of the clinically significant TG/HDL ratio varied in different populations [14].

Nevertheless, some studies revealed why a better biomarker, such as AIP, is needed to predict cardiovascular disease (CVD) or obesity compared with conventional biomarkers. For instance, abnormally increased LDL-C levels indeed indicate higher risk of coronary artery disease (CAD) events. However, many CVD patients do not exhibit elevated LDL-C levels. In Framingham cohort study, a geographically defined population-based study enrolling 374 subjects with a history of myocardial infarction, showed that 20% of myocardial infarction events occurred in patients whose average total cholesterol and LDL-C levels were lower than the recommended levels according to the guidelines of the National Cholesterol Education Program [15,16]. Therefore, it is not sufficient to use merely conventional lipid profiles to predict CVD or obesity.

The high CVD predictive value of AIP might be explained due to its positive relation to small dense LDL (sdLDL). It has been found that AIP is closely related to the size of lipoprotein particle and FERHDL [10,17]. Similar result was also discovered from an analysis of results of 11 previous studies [18].

Circulating lipoprotein particles can be classified into many categories according to its size and density. Small dense LDL is a subclass of LDL separated by ultracentrifugation or gradient gel electrophoresis from other LDL [19]. Compared to LDL, sdLDL is susceptible to oxidation and responsible for plaque formation, which induce atherosclerotic lesions. Therefore, sdLDL was identified as a risk factor for cardiovascular events by the National Cholesterol Education Program [20].

The size of low density lipoprotein can be reflected by the Fractional esterification rate of cholesterol in high-density lipoprotein (FERHDL) [21]. FERHDL is defined as the percentage of HDL free cholesterol esterified during HDL incubation. Junmeng Liu et al. have documented significant relations between FERHDL and lipid profiles, including the positive correlation with sdLDL concentrations in healthy subjects [22].

The association among ADMA, FABP4 and AIP in a cross-sectional study of 340 healthy women may also explain why AIP could be a biomarker for cardiovascular disease [23]. Asymmetric dimethylarginine (ADMA) competes with the substrate of L-arginine and inhibits vascular NO production. Thus, further endothelial dysfunction and subsequent atherosclerosis is induced [24]. Fatty acid-binding protein 4 (FABP4) is mainly existed in macrophages and adipocytes. Increased FABP4 induces lipolysis in the adipocytes and contributes to insulin resistance and inflammation [25,26,27]. Accordingly, FABP4 as well as ADMA is novel cardiovascular risk factor, and AIP could also play an emerging role due to its accessibility and inexpensiveness.

To date, many studies have suggested diverse applications of AIP. It has been found that AIP is associated with obesity in a large-sample cross-sectional study in China. The results of their study showed that AIP exhibited a strong association with obesity with an adjusted odds ratio of 5.55 [5]. Our study, which reported an adjusted odds ratio of 3.22, also indicated the close correlation between AIP and obesity. Besides, in a study conducted by Ping Song et al., AIP indicated not only high BMI but also high body fat level and visceral fat area in type 2 diabetes mellitus patients [28].

Our study has some advantages. First, there are not many studies about AIP and obesity in Taiwan; therefore, the regional data and analysis in our study are needed. In addition, this survey was performed in the same hospital that has a strict and standard examination process. The predictive model of our study is also reliable based on rigorous analysis with significant results.

There are also some limitations in the study. First, the cross-sectional study did not reveal a causal relationship between AIP and obesity. Further cohort studies with follow-up data are needed to verify the results. Second, the study only included participants who were working in the hospital; therefore, these findings may not be generalized to general population in Taiwan. Third, potential confounders, especially those that could affect obesity, such as lifestyle and dietary patterns, were not included in our study. Finally, some information was unavailable from the data used in this study, such as history of endocrine disease or drug usage, which potentially influenced obesity.

## 5. Conclusions

In conclusion, higher AIP levels were strongly associated with the presence of obesity in Taiwanese hospital employees. However, the causal relationship between AIP and obesity is still unknown. Further cohort studies are needed to discover the mechanism.

## Figures and Tables

**Table 1:** Clinical characteristics between obesity and non-obesity group among the study population.

Variables	Total	Non-Obesity (BMI < 27)	Obesity (BMI ≥ 27)	*p* Value
	(*n* = 1312)	(*n* = 989)	(*n* = 323)	
Age (year)	42.39 ± 8.90	42.20 ± 9.03	42.97 ± 8.47	0.18
SBP (mmHg)	123.06 ± 16.09	120.01 ± 14.97	132.39 ± 15.83	<0.001
DBP (mmHg)	76.38 ± 12.39	74.09 ± 11.53	83.39 ± 12.31	<0.001
BMI (kg/m^2^)	24.50 ± 4.11	22.66 ± 2.46	30.13 ± 2.85	<0.001
WC (cm)	81.01 ± 10.55	76.97 ± 7.70	93.38 ± 8.22	<0.001
ALT (U/L)	25.74 ± 21.99	21.86 ± 17.52	37.63 ± 28.95	<0.001
FPG (mg/dL)	90.36 ± 18.84	88.13 ± 13.73	97.20 ± 28.37	<0.001
HDL-C (mg/dL)	55.73 ± 13.28	58.13 ± 13.05	48.37 ± 11.10	<0.001
LDL-C (mg/dL)	116.02 ± 29.33	113.29 ± 28.52	124.29 ± 30.22	<0.001
TC (mg/dL)	190.68 ± 32.46	189.57 ± 31.73	194.07 ± 34.45	0.03
TG (mg/dL)	99.19 ± 70.37	88.32 ± 61.06	132.46 ± 85.14	<0.001
Men, *n* (%)	501 (38.19%)	341 (34.48%)	160 (49.54%)	<0.001
HTN, *n* (%)	293 (22.33%)	149 (15.07%)	144 (44.58%)	<0.001
DM, *n* (%)	62 (4.73%)	32 (3.24%)	30 (9.29%)	<0.001
Hyperlipidemia, *n* (%)	603 (45.96%)	411 (41.56%)	192 (59.44%)	<0.001
Metabolic syndrome, *n* (%)	223 (17.00%)	59 (5.97%)	164 (50.77%)	<0.001

Abbreviations: SBP, systolic blood pressure; DBP diastolic blood pressure; BMI, body mass index; WC, waist circumstance; ALT, alanine aminotransferase; FPG, fasting plasma glucose; HDL-C, high-density lipoprotein cholesterol; LDL-C, low-density lipoprotein cholesterol; TC, total cholesterol; TG, triglyceride; HTN, hypertension; DM, diabetes mellitus. (Note: Clinical characteristics are expressed as the mean ± standard deviation for continuous variables and *n* (%) for categorical variables. *p*-value were derived from independent *t*-test for continuous variables and chi-square test for categorical variables).

**Table 2:** Clinical characteristics according to AIP tertiles among the study population.

Variables	AIP				
	Total	Low (<−0.3387)	Middle (−0.3387, −0.059)	High (>−0.059)	*p* Value
	(*n* = 1312)	(*n* = 437)	(*n* = 438)	(*n* = 437)	
Age (year)	42.39 ± 8.90	39.84 ± 8.38	42.79 ± 8.72 ^a^	44.53 ± 8.98 ^a,b^	<0.001
SBP (mmHg)	123.06 ± 16.09	116.59 ± 14.61	122.52 ± 15.66 ^a^	130.08 ± 15.08 ^a,b^	<0.001
DBP (mmHg)	76.38 ± 12.39	71.06 ± 10.55	75.48 ± 11.69 ^a^	82.61 ± 12.04 ^a,b^	<0.001
BMI (kg/m^2^)	24.50 ± 4.11	22.30 ± 3.30	24.39 ± 3.91 ^a^	26.81 ± 3.80 ^a,b^	<0.001
WC (cm)	81.01 ± 10.55	74.36 ± 7.81	80.58 ± 9.49 ^a^	88.08 ± 9.42 ^a,b^	<0.001
ALT (U/L)	25.74 ± 21.99	17.64 ± 9.65	23.52 ± 16.27 ^a^	36.08 ± 30.29 ^a,b^	<0.001
FPG (mg/dL)	90.36 ± 18.84	85.63 ± 10.98	88.84 ± 11.86 ^a^	96.61 ± 27.25 ^a,b^	<0.001
HDL-C (mg/dL)	55.73 ± 13.28	67.08 ± 11.30	55.55 ± 9.21 ^a^	44.56 ± 7.95 ^a,b^	<0.001
LDL-C (mg/dL)	116.02 ± 29.33	103.06 ± 24.47	117.09 ± 26.24 ^a^	127.75 ± 31.47 ^a,b^	<0.001
TC (mg/dL)	190.68 ± 32.46	184.81 ± 30.75	188.63 ± 30.11	198.59 ± 34.82 ^a,b^	<0.001
TG (mg/dL)	99.19 ± 70.37	49.33 ± 12.05	81.05 ± 16.10 ^a^	167.22 ± 83.82 ^a,b^	<0.001
Men, *n* (%)	501 (38.19%)	55 (12.59%)	162 (36.99%) ^a^	284 (64.99%) ^a,b^	<0.001
HTN, *n* (%)	293 (22.33%)	37 (8.47%)	79 (18.04%) ^a^	177 (40.50%) ^a,b^	<0.001
DM, *n* (%)	62 (4.73%)	8 (1.83%)	12 (2.74%)	42 (9.61%) ^a,b^	<0.001
Hyperlipidemia, *n* (%)	603 (45.96%)	134 (30.66%)	165 (37.67%)	304 (69.57%) ^a,b^	<0.001
Metabolic syndrome, *n* (%)	223 (17.00%)	5 (1.14%)	29 (6.62%) ^a^	189 (43.25%) ^a,b^	<0.001

Abbreviations: AIP, atherogenic index of plasma; SBP, systolic blood pressure; DBP diastolic blood pressure; BMI, body mass index; WC, waist circumstance; ALT, alanine aminotransferase; FPG, fasting plasma glucose; HDL-C, high-density lipoprotein cholesterol; LDL-C, low-density lipoprotein cholesterol; TC, total cholesterol; TG, triglyceride; HTN, hypertension; DM, diabetes mellitus. (Note: Clinical characteristics are expressed as the mean ± standard deviation for continuous variables and *n* (%) for categorical variables. *p*-value were derived from one-way ANOVA for continuous variables and chi-square test for categorical variables). ^a^ *p* < 0.05 vs. AIP lower group; ^b^ *p* < 0.05 vs. AIP middle group in the Bonferroni post hoc comparisons.

**Table 3:** Logistic regression analysis for obesity among study population.

Variables	Unadjusted OR	(95% CI)	*p* Value	Adjusted OR	(95% CI)	*p* Value
Age (year)	1.01	(0.996–1.02)	0.18	0.97	(0.96–0.99)	0.005
Sex (men versus women)	1.87	(1.45–2.41)	<0.001	0.54	(0.38–0.76)	<0.001
HTN (yes versus no)	4.54	(3.43–6.00)	<0.001	3.74	(2.65–5.30)	<0.001
DM (yes versus no)	3.06	(1.83–5.12)	<0.001	1.85	(0.99–3.49)	0.06
HDL-C (mg/dL)	0.93	(0.92–0.94)	<0.001	1.00	(0.97–1.03)	0.97
TC (mg/dL)	1.00	(1.00–1.01)	0.03	0.96	(0.94–0.99)	0.002
TG (mg/dL)	1.01	(1.007–1.01)	<0.001	1.01	(1.00–1.01)	0.005
LDL-C (mg/dL)	1.01	(1.01–1.02)	<0.001	1.05	(1.02–1.07)	<0.001
AIP						
Low	1.00	-	-	-	-	-
Middle	3.79	(2.48–5.79)	<0.001	2.31	(1.41–3.78)	0.001
High	9.74	(6.49–14.62)	<0.001	3.22	(1.71–6.07)	<0.001

Abbreviations: CI, confidence interval; OR, odds ratio; HTN, hypertension; DM, diabetes mellitus; HDL-C, high-density lipoprotein cholesterol; TC, total cholesterol; TG, triglyceride; LDL-C, low-density lipoprotein cholesterol; AIP, atherogenic index of plasma. (Note: 1. obesity = BMI ≥ 27 kg/m^2^; 2. for adjusted OR, all variables in the table were adjusted).

**Table 4:** Area under the ROC curve (95% CI) for obesity according to three lipid components and AIP.

Variables	AUC (95% CI)	*p* Value	Cut-Off Point	Sensitivity	Specificity
TC (mg/dL)	0.53 (0.50–0.57)	0.07	200.50	0.41	0.66
TG (mg/dL)	0.71 (0.68–0.74)	<0.001	82.50	0.75	0.60
LDL-C (mg/dL)	0.61 (0.57–0.64)	<0.001	116.50	0.58	0.58
AIP	0.74 (0.70–0.77)	<0.001	−0.16	0.74	0.63

Abbreviations: ROC curve, receiver operating characteristic curve; CI, confidence interval; TC, total cholesterol; TG, triglyceride; LDL-C, low-density lipoprotein cholesterol; AIP, atherogenic index of plasma.

## Data Availability

The data presented in this study are available on request from the corresponding author.

## References

[1] Blüher M. (2019). Obesity: Global epidemiology and pathogenesis. Nat. Rev. Endocrinol..

[2] Bays H.E., González-Campoy J.M., Bray G.A., Kitabchi A.E., Bergman D.A., Schorr A.B., Rodbard H.W., Henry R.R. (2008). Pathogenic potential of adipose tissue and metabolic consequences of adipocyte hypertrophy and increased visceral adiposity. Expert Rev. Cardiovasc. Ther..

[3] Bays H., Mandarino L., DeFronzo R.A. (2004). Role of the adipocyte, free fatty acids, and ectopic fat in pathogenesis of type 2 diabetes mellitus: Peroxisomal proliferator-activated receptor agonists provide a rational therapeutic approach. J. Clin. Endocrinol. Metab..

[4] Bays H.E., Toth P.P., Kris-Etherton P.M., Abate N., Aronne L.J., Brown W.V., Gonzalez-Campoy J.M., Jones S.R., Kumar R., La Forge R. (2013). Obesity, adiposity, and dyslipidemia: A consensus statement from the National Lipid Association. J. Clin. Lipidol..

[5] Zhu X., Yu L., Zhou H., Ma Q., Zhou X., Lei T., Hu J., Xu W., Yi N., Lei S. (2018). Atherogenic index of plasma is a novel and better biomarker associated with obesity: A population-based cross-sectional study in China. Lipids Health Dis..

[6] Yeh T.-L., Chen H.-H., Chiu H.-H., Chiu Y.-H., Hwang L.-C., Wu S.-L. (2019). Morbidity associated with overweight and obesity in health personnel: A 10-year retrospective of hospital-based cohort study in Taiwan. Diabetes Metab. Syndr. Obes..

[7] Yeh W.-C., Chuang H.-H., Lu M.-C., Tzeng I.S., Chen J.-Y. (2018). Prevalence of metabolic syndrome among employees of a taiwanese hospital varies according to profession. Medicine.

[8] Dobiásová M. (2004). Atherogenic index of plasma [log(triglycerides/HDL-cholesterol)]: Theoretical and practical implications. Clin. Chem..

[9] Dobiásová M. (2006). [AIP--atherogenic index of plasma as a significant predictor of cardiovascular risk: From research to practice]. Vnitr. Lek..

[10] Frohlich J., Dobiásová M. (2003). Fractional esterification rate of cholesterol and ratio of triglycerides to HDL-cholesterol are powerful predictors of positive findings on coronary angiography. Clin. Chem..

[11] Niroumand S., Khajedaluee M., Khadem-Rezaiyan M., Abrishami M., Juya M., Khodaee G., Dadgarmoghaddam M. (2015). Atherogenic Index of Plasma (AIP): A marker of cardiovascular disease. Med. J. Islamic Repub. Iran.

[12] Zhang X., Zhang X., Li X., Feng J., Chen X. (2019). Association of metabolic syndrome with atherogenic index of plasma in an urban Chinese population: A 15-year prospective study. Nutr. Metab. Cardiovasc. Dis..

[13] McLaughlin T., Abbasi F., Cheal K., Chu J., Lamendola C., Reaven G. (2003). Use of metabolic markers to identify overweight individuals who are insulin resistant. Ann. Intern. Med..

[14] McLaughlin T., Reaven G., Abbasi F., Lamendola C., Saad M., Waters D., Simon J., Krauss R.M. (2005). Is there a simple way to identify insulin-resistant individuals at increased risk of cardiovascular disease?. Am. J. Cardiol..

[15] Wong N.D., Wilson P.W., Kannel W.B. (1991). Serum cholesterol as a prognostic factor after myocardial infarction: The Framingham Study. Ann. Intern. Med..

[16] Kannel W.B. (1995). Range of serum cholesterol values in the population developing coronary artery disease. Am. J. Cardiol..

[17] Dobiášová M., Frohlich J. (2001). The plasma parameter log (TG/HDL-C) as an atherogenic index: Correlation with lipoprotein particle size and esterification rate inapob-lipoprotein-depleted plasma (FERHDL). Clin. Biochem..

[18] Dobiásová M., Frohlich J. (2000). [The new atherogenic plasma index reflects the triglyceride and HDL-cholesterol ratio, the lipoprotein particle size and the cholesterol esterification rate: Changes during lipanor therapy]. Vnitr. Lek..

[19] Ivanova E.A., Myasoedova V.A., Melnichenko A.A., Grechko A.V., Orekhov A.N. (2017). Small Dense Low-Density Lipoprotein as Biomarker for Atherosclerotic Diseases. Oxidative Med. Cell. Longev..

[20] (2002). Third Report of the National Cholesterol Education Program (NCEP) Expert Panel on Detection, Evaluation, and Treatment of High Blood Cholesterol in Adults (Adult Treatment Panel III) final report. Circulation.

[21] Ohta T., Saku K., Takata K., Nagata N., Maung K.K., Matsuda I. (1997). Fractional esterification rate of cholesterol in high density lipoprotein (HDL) can predict the particle size of low density lipoprotein and HDL in patients with coronary heart disease. Atherosclerosis.

[22] Liu J., Yang R., Zhou M., Mao W., Li H., Zhao H., Wang S., Chen W., Dong J., He Q. (2017). Fractional esterification rate of cholesterol in high-density lipoprotein associates with risk of coronary heart disease. Lipids Health Dis..

[23] Fernández-Macías J.C., Ochoa-Martínez A.C., Varela-Silva J.A., Pérez-Maldonado I.N. (2019). Atherogenic Index of Plasma: Novel Predictive Biomarker for Cardiovascular Illnesses. Arch. Med. Res..

[24] Böger R.H. (2003). The emerging role of asymmetric dimethylarginine as a novel cardiovascular risk factor. Cardiovasc. Res..

[25] Dou H.-X., Wang T., Su H.-X., Gao D.-D., Xu Y.-C., Li Y.-X., Wang H.-Y. (2020). Exogenous FABP4 interferes with differentiation, promotes lipolysis and inflammation in adipocytes. Endocrine.

[26] Lee C.H., Lam K.S. (2019). Obesity-induced insulin resistance and macrophage infiltration of the adipose tissue: A vicious cycle. J. Diabetes Investig..

[27] Lee C.-H., Lui D.T.W., Lam K.S.L. (2021). Adipocyte Fatty Acid-Binding Protein, Cardiovascular Diseases and Mortality. Front. Immunol..

[28] Song P., Xu L., Xu J., Zhang H.Q., Yu C.X., Guan Q.B., Zhao M., Zhang X. (2018). Atherogenic Index of Plasma is Associated with Body Fat Level in Type 2 Diabetes Mellitus Patients. Curr. Vasc. Pharm..

